# Development and Validation of a Simple Risk Score for Undiagnosed Type 2 Diabetes in a Resource-Constrained Setting

**DOI:** 10.1155/2016/8790235

**Published:** 2016-09-04

**Authors:** Antonio Bernabe-Ortiz, Liam Smeeth, Robert H. Gilman, Jose R. Sanchez-Abanto, William Checkley, J. Jaime Miranda, CRONICAS Cohort Study Group

**Affiliations:** ^1^CRONICAS Center of Excellence in Chronic Diseases, Universidad Peruana Cayetano Heredia, Lima, Peru; ^2^Faculty of Epidemiology and Population Health, London School of Hygiene and Tropical Medicine, London, UK; ^3^Department of International Health, Bloomberg School of Public Health, Johns Hopkins University, Baltimore, MD, USA; ^4^Área de Investigación y Desarrollo, Asociación Benéfica PRISMA, Lima, Peru; ^5^Centro Nacional de Alimentación y Nutrición, Instituto Nacional de Salud, Lima, Peru; ^6^Division of Pulmonary and Critical Care, School of Medicine, Johns Hopkins University, Baltimore, MD, USA; ^7^Department of Medicine, School of Medicine, Universidad Peruana Cayetano Heredia, Lima, Peru; ^8^Universidad Peruana Cayetano Heredia, Lima, Peru

## Abstract

*Objective*. To develop and validate a risk score for detecting cases of undiagnosed diabetes in a resource-constrained country.* Methods*. Two population-based studies in Peruvian population aged ≥35 years were used in the analysis: the ENINBSC survey (*n* = 2,472) and the CRONICAS Cohort Study (*n* = 2,945). Fasting plasma glucose ≥7.0 mmol/L was used to diagnose diabetes in both studies. Coefficients for risk score were derived from the ENINBSC data and then the performance was validated using both baseline and follow-up data of the CRONICAS Cohort Study.* Results*. The prevalence of undiagnosed diabetes was 2.0% in the ENINBSC survey and 2.9% in the CRONICAS Cohort Study. Predictors of undiagnosed diabetes were age, diabetes in first-degree relatives, and waist circumference. Score values ranged from 0 to 4, with an optimal cutoff ≥2 and had a moderate performance when applied in the CRONICAS baseline data (AUC = 0.68; 95% CI: 0.62–0.73; sensitivity 70%; specificity 59%). When predicting incident cases, the AUC was 0.66 (95% CI: 0.61–0.71), with a sensitivity of 69% and specificity of 59%.* Conclusions*. A simple nonblood based risk score based on age, diabetes in first-degree relatives, and waist circumference can be used as a simple screening tool for undiagnosed and incident cases of diabetes in Peru.

## 1. Introduction

As of 2014, the worldwide prevalence of type 2 diabetes mellitus (T2DM) was estimated to be 9% among adults aged ≥18 years with great impact on mortality, particularly in low- and middle-income countries (LMIC) [1, 2]. Moreover, globally, approximately 25% to 75% of diabetes cases remain undiagnosed [3, 4], until further complications, especially at the macro- and micro-vascular level, manifest clinically. In Latin America, the proportion of undiagnosed diabetes at the population level ranged from 33% to 50% [5].

An important strategy to prevent or delay T2DM complications is the early identification of those with undiagnosed diabetes; yet, universal screening for diabetes at the population level is not practical in resource-limited settings. The American Diabetes Association recommends the use of glucose test as T2DM screening in people with overweight and obesity as well as in those with other risk factors [6]. As a result, risk assessment scores have been developed to address this problem in a simple and inexpensive way. Most of the available algorithms for diabetes screening have been developed in Caucasian [7–9] and Asian populations [10–13] and very few in other ethnic groups [14, 15]. To date, one diabetes risk score has been developed and validated in Latin America so far which was derived from one urban area in Brazil [16], thus bearing limited generalizability to the wider region. Furthermore, it is well established that before adopting existing risk scores as screening tools in different populations and ethnic groups, their performance needs to be evaluated, calibrated, or validated in local settings [17].

As the American Diabetes Association, the Peruvian Ministry of Health recommends diabetes screening in general population with fasting glucose in adults aged 40 to 70 years with risk factors. However, fasting glucose is not always available in primary care settings, especially in semiurban and rural areas. As a result, a major challenge to be overcome in many countries is the implementation of a simple, fast, and laboratory-free based screening method.

Consequently, we aimed to develop a simple laboratory-free risk score to identify people with undiagnosed diabetes and incident diabetes in Peru, a Latin American country that spans coastal, Andean, and rainforest settings. In order to do so, this work benefited from two large-scale population-based surveys: the first one, representative at the national level, was used to develop the score, and the second one, a cohort study, was utilized for external validation.

## 2. Methods

### 2.1. Study Design and Participants

Two different population-based studies were used in this analysis. The* National Survey of Nutritional and Biochemical Indicators for Noncommunicable Diseases* (ENINBSC in Spanish), conducted by the Peruvian National Institute of Health [18], was used to develop our predictive model. This was complemented with the CRONICAS Cohort Study [19], whose baseline and longitudinal information was used to validate the risk score.

The ENINBSC is a national population-based survey carried out in Peru between August 2004 and April 2005, designed to estimate the prevalence of hypertension, type 2 diabetes mellitus, and other risk factors for noncommunicable diseases at the national and regional level [18]. Potential participants were those aged ≥20 years, habitual residents in the study area, and able to provide consent for their participation in the study. Pregnant women and those currently breastfeeding were excluded from the study. As per design, the ENINBSC sample was stratified according to Peru's five major regions of the country: Lima, rest of the Coast, urban Highlands, rural Highlands, and jungle. In each stratum, cluster of blocks were chosen using single random sampling techniques. Within each cluster, a random sample of households and participants were selected.

The CRONICAS Cohort Study is an ongoing cardiopulmonary project aimed to estimate the prevalence and incidence of hypertension, diabetes mellitus, and obesity in four different settings in Peru that differ in terms of their urbanicity and altitude: Pampas de San Juan de Miraflores, in the highly urbanized Lima, Puno in the altitude (3,825 meters above the sea level) contributing with rural and urban areas, and Tumbes, a semiurban area in the northern coast of Peru [19]. The study started in September 2010 and a follow-up visit was completed in March 2014. A sex- and age-stratified sample was selected at random for each of the settings and all participants aged ≥35 years, full time residents in the study area, and able to consent, were enrolled. Follow-up data used for this analysis was collected, on average, at 30 months after baseline.

### 2.2. Study Procedures

The procedures of the ENINBSC have been described previously [18]. Briefly, after consent, two different visits were scheduled. The first one lasted on average 40 minutes and was carried out to apply a face-to-face questionnaire regarding data about household characteristics, demographics, lifestyles behaviors, risk factors, and blood pressure measurements. The second visit lasted 30 minutes on average and was planned to have an appropriate period of fasting for blood sampling for glucose, total cholesterol, HDL-cholesterol, and the remaining anthropometric measures (height, weight, and waist circumference) using standard procedures.

Similarly, the procedures of the CRONICAS study has been published elsewhere [19]. In brief, participants responded to a face-to-face questionnaire applied by trained community health workers. Data collected comprised risk factors for cardiovascular disease based on a modified version of the WHO STEP approach questionnaire for surveillance of noncommunicable disease [20]. A period of 8 to 12 hours of fasting was required for blood sampling to collect fasting glucose, total cholesterol, and HDL-cholesterol. Height, weight, and waist circumference were also assessed, and blood pressure was measured in triplicate after five minutes of resting using an automatic monitor (OMRON HEM-780) previously validated in adult's population [21].

### 2.3. Variable Definitions

In both studies, diabetes was defined as any of the following conditions: fasting glucose ≥7.0 mmol/L (≥126 mg/dL) and/or self-report of physician diagnosis. Fasting glucose was assessed by an enzymatic colorimetric method (glucose oxidase GOD-PAP) in both studies. After excluding individuals without known diabetes, undiagnosed diabetes was also estimated to develop and validate the risk score [22].

Variables included in the analyses were built to guarantee similarities between both studies: sex; age (<55 and ≥55 years); education (in years); self-reported smoking (current versus never/former smoker); alcohol use (user versus never user); self-reported diabetes in first-degree relatives (participant's parents and/or siblings); and levels of physical activity (low versus moderate/high levels, based on the transport-related domain of the IPAQ). Anthropometric measurements included in the analysis were body mass index ((BMI), <25, 25–29.9, and ≥30 Kg/m^2^), waist circumference (<90, 90–99.9, and ≥100 cm), waist-to-height ratio (<0.50, 0.50–0.59, 0.60–0.69, and ≥0.70) [23], and hypertension (measured or previously diagnosed) [24].

### 2.4. Statistical Analysis

A total of 4,206 participants were enrolled in the ENINBSC, but only 2,472 were included in this analysis. Reasons for exclusion were 1,524 because of age <35 years to make both databases comparable, 129 because of no data about fasting plasma glucose levels being available, and 81 because of known diagnosis of diabetes. In the CRONICAS study, 3,601 participants were enrolled at baseline but only 2,948 records were analyzed as 465 had no data about glucose levels, and 188 were excluded because of previous diagnosis of diabetes. In addition, only data from 2,577 participants was used in the longitudinal assessment of the risk score (comparison of baseline characteristics among those included and excluded from longitudinal analysis is shown in Online Supplement: E-Table 1; see Supplementary Material available online at http://dx.doi.org/10.1155/2016/8790235).

Initially, population characteristics of both studies were tabulated using proportions in the case of categorical variables and means and standard deviation (SD) with numerical variables. Then, the prevalence and 95% confidence intervals (95% CI) of total diabetes and undiagnosed diabetes were estimated in each study. After that, all cases of known diabetes were excluded from subsequent analyses.

The risk score was derived from data of the ENINBSC survey taking into account the multistage sampling strategy of the study. Each potential risk factor (i.e., sex, age, family history of diabetes, etc.) was assessed in bivariate models using logistic regression and undiagnosed diabetes as the dependent variable. Then, risk factors with a *p* value <0.10 in the bivariate analysis were included in a multiple logistic regression model using stepwise backward elimination with a significance level of 5%. The Hosmer-Lemeshow goodness-of-fit test was used to assess how well the predicted prevalence matched the observed prevalence of undiagnosed diabetes (i.e., *p* values over 0.20 indicate that model fits well) [25]. As we sought for an easily applicable and implementable algorithm, the risk factors in the final model were each assigned a weighted score by rounding up all regression coefficients in the final model to the nearest integer as in a previous report [26].

For the evaluation of the risk score, the area under the receiver operating characteristic (ROC) curve, sensitivity, specificity, and positive and negative predictive values (PPV and NPV) were calculated. The optimal cut-point was determined using the Youden index, a single statistic that captures the performance of a diagnostic test (i.e., sensitivity + specificity − 1) [27]. As one of the main aims of a nonlaboratory risk score is to identify people who warrant having a blood test (i.e., fasting glucose, glycated haemoglobin, etc.), the cut-point with the highest sensitivity was also estimated and described.

We assessed the performance of our score using bootstrap techniques as well as carrying out an external validation using the CRONICAS Cohort Study. Bootstrapping was utilized to estimate confidence intervals for the AUC in our study population. A total of 1,000 random samples with replacement were taken from the development database. The resulting 1,000 prediction models were then assessed to estimate the bootstrap AUC using the bias-corrected version of the confidence intervals [28]. In addition, using baseline data from the CRONICAS Cohort Study, validation measures (AUC, sensitivity, specificity, predictive values, and likelihood ratios) were estimated.

To evaluate the performance of our algorithm, the Peruvian risk score was compared to previously published models for undiagnosed diabetes including the Brazilian risk score [16], the Qingdao score [10], the Indian risk score [11], the Kuwaiti risk score [29], the patient self-assessment score [26], and the Rotterdam risk score [7] using the c-statistic. Finally, using the follow-up data of the CRONICAS Cohort Study, the risk score was also evaluated to detect incident cases of T2DM by excluding those with diabetes diagnosis at baseline. Analyses were performed using STATA 13.0 (StataCorp, College Station, TX, USA).

### 2.5. Ethical Issues

The protocol and informed consent forms of the ENINBSC study were reviewed and approved by the* Instituto Nacional de Salud* and the* Centro Nacional de Alimentación y Nutrición*, both part of the Ministry of Health in Lima, Peru. In the case of the CRONICAS Cohort Study, protocol and consent forms were reviewed and approved by the institutional review boards of the Universidad Peruana Cayetano Heredia and the NGO Asociación Benéfica PRISMA in Lima, Peru, and the Johns Hopkins University in Baltimore, USA.

## 3. Results

The characteristics of participants in both studies are detailed in Table 1. Overall, participants from the CRONICAS study were 5 years older, reported consuming lower levels of alcohol, and were less physically active than those from the ENINBSC survey.

### 3.1. Prevalence of Diabetes and Undiagnosed Diabetes

The overall prevalence of diabetes was 5.1% (129/2538; 95% CI: 4.2%–5.9%) in the ENINBSC survey and 8.7% (272/3135; 95% CI: 7.7%–9.7%) in the CRONICAS Cohort Study's baseline. After excluding those with known diabetes, undiagnosed diabetes was present in 2.0% (48/2457; 95% CI: 1.4%–2.5%) in the ENINBSC survey and in 2.9% (85/2948; 95% CI: 2.3%–3.5%) in the CRONICAS Cohort Study.

### 3.2. Development of the Risk Score

**Figure 1:**
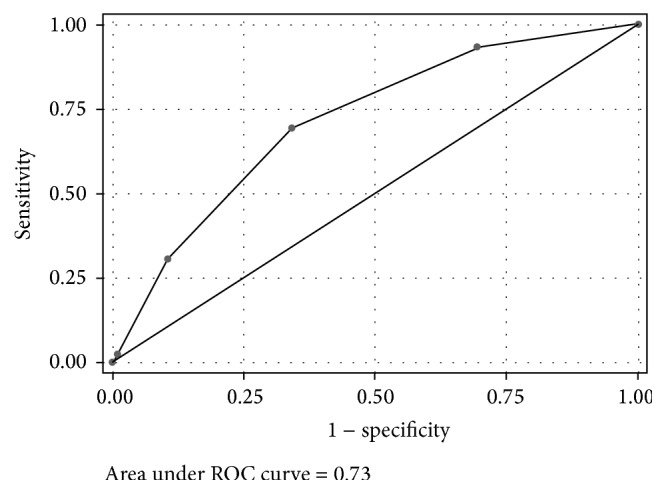
Receiver operating characteristic (ROC) curve of the risk score in predicting undiagnosed type 2 diabetes in the development database. The area under the ROC curve was 0.73 (95% CI: 0.65–0.78) for the risk score.

After stepwise backward logistic regression, age, diabetes in first-degree relatives, and waist circumference were independently associated with undiagnosed diabetes (Table 2). The Hosmer-Lemeshow test showed that the final model fitted relatively well (*p* = 0.21). The Peruvian diabetes risk score was constructed based on the coefficients of that final regression model. The score gave an AUC of 0.73 (95% CI: 0.65–0.78), and the optimal cut-point for undiagnosed diabetes using the Youden index was ≥2 (Figure 1). With this cut-point, about 34.8% of participants were categorized as at high risk of diabetes: sensitivity 69.6%, specificity 65.8%, and PPV and NPV of 3.9% and 99.1%, respectively. With a cut-point ≥1, 69.8% of participants would be at high risk of diabetes with improved sensitivity (93.5%) but lower specificity (30.6%).  Table 3 shows the performance of the risk score for detecting undiagnosed diabetes at different cut-points.

### 3.3. Cross-Sectional Validation of the Risk Score

When bootstrap was used, the performance of our risk score was similar to the obtained in the development model (AUC = 0.72; 95% CI: 0.65–0.78). In addition, when the risk score was evaluated by applying the score to the CRONICAS Cohort Study's population, the AUC for undiagnosed diabetes was 0.68 (95% CI: 0.62–0.73). At the suggested cut-point of ≥2, 42% would be categorized as undiagnosed diabetes with sensitivity, specificity, PPV, and NPV of 70.2%, 58.9%, 4.8%, and 98.5%, respectively (Table 4). On the other hand, with a cut-point ≥1, 80% would be categorized as undiagnosed diabetes with sensitivity, specificity, PPV, and NPV of 94.0%, 20.0%, 3.3%, and 99.1%, respectively.

When previous published algorithms for undiagnosed diabetes were applied to the CRONICAS Cohort Study, the performance of the Rotterdam score (*p* < 0.001), Indian score (*p* < 0.001), and Qingdao score (*p* < 0.01) was poorer than our score; however, our algorithm performed similar to the other assessed models, such as the Brazilian risk score (*p* = 0.93), the Kuwaiti score (*p* = 0.26), and the patient self-assessment score (*p* = 0.74), but having only three variables.

### 3.4. Longitudinal Assessment of the Risk Score

The performance of this risk score was also assessed to predict incident cases of diabetes using the longitudinal data from the CRONICAS Cohort Study. One hundred twenty-one new cases of diabetes were found accounting for 6,207 person-years at risk, with an overall incidence of 1.95 (95% CI: 1.63–2.33) cases per 100 person-years of risk. The AUC of the score was 0.66 (95% CI: 0.61–0.71). With a cut-point ≥2, 42.5% of participants were categorized as at high risk of developing diabetes: sensitivity, specificity, PPV, and NPV were 69.4%, 58.9%, 7.8%, and 97.4%, whereas, for a cut-point ≥1, the respective values were 79.9%, 91.9%, 20.7%, 5.5%, and 98.1%.

## 4. Discussion

### 4.1. Main Findings

Using a national population-based survey, a simple nonblood based risk score based on age, history of diabetes in first-degree relatives, and waist circumference was built and shown to perform moderately in detecting undiagnosed diabetes when externally validated. Moreover, the performance of the score was almost similar for detecting incident cases of diabetes in the Peruvian population.

### 4.2. Comparison with Other Risk Scores

A relatively recent systematic literature search found 23 different blood-free prevalent diabetes risk scores: ten from Europe, nine for Asian populations, two from the United States, and two from Middle East [30]. In addition, and not included in the aforementioned review, only one risk score was developed in Latin America using Brazilian urban population [16]. The same systematic review reported that AUC for these predictive models was greater in the development studies (range: 0.65 to 0.88) than in the validation studies (range: 0.63 to 0.80) [30], similar to our findings. Another systematic review found that several noninvasive algorithms were created using variables such as age, gender, waist circumference and/or BMI, and family history of diabetes in the final model [31]. As impracticality due to use of the algorithms was a common barrier to the uptake of risk scores by healthcare staff and individuals [32], our model, created with three of these more common variables, reached a moderate-to-high sensitivity depending on the used cut-point. Moreover, two of these variables are easily evaluable during medical appointment or through individual's self-assessment, and only a measuring tape and no calculations are required to be implemented in clinical practice or at the population level.

From a cross-sectional point of view, with a cut-point ≥2, from 1000 participants assessed by the Peruvian diabetes risk score, a total of 420 would be classified as undiagnosed diabetes with the detection of 20 cases and only 6 will be missing. On the other hand, with a cut-point ≥1, from 1000 screened individuals, a total of 804 would be categorized as having undiagnosed diabetes with the detection of 27 cases and only 7 will be missing. Thus, the reduction of the cut-point of the risk score would increase sensitivity but reducing the specificity and imposing the need of performing a confirmatory test (i.e., fasting glucose) to almost the double of individuals, with the benefit of having only 7 more people diagnosed.

Longitudinally, the same risk score would detect an important number of participants at risk of developing diabetes: 43% of screened individuals would be classified at high risk of diabetes, and of them, 8% would develop diabetes in the next 2.5 years. According to a previous study [33], 17 reports described a noninvasive model to predict the development of diabetes and included a median of six risk predictors, ranging from 2 to 11 [34]. Although our score did not perform as good as other well-known longitudinal models in the literature such as the FINDRISC or the ARIC scores [35, 36], it only included three variables and was built using cross-sectional information. In addition, some variables used in the aforementioned studies are difficult to standardize within a country as Peru, that is, food portions, physical activity, or sedentarism, limiting therefore its use on a wider scale and in a simple pragmatic fashion.

Our algorithm performed better than the Rotterdam, the Indian, and the Qingdao risk scores in our population, which highlights the need of calibration and/or development of a specific score for different ethnic groups before its adoption. As there are ethnic differences in risk factors for diabetes and Peru is considered a multiethnic country, it is necessary to create specific scores or recalibrate existing algorithms before applying in specific contexts. In addition, with only three variables included, the performance of our predictive model was similar to the other assessed scores included in the analyses. Taken together, the score developed has the potential to augment, in a pragmatic manner, initial rapid screening for diabetes, especially at various nonspecialized primary healthcare services.

Our findings also demonstrate that approximately 35% of cases of T2DM (39% in the ENINBSC survey and 33% in the baseline of the CRONICAS Cohort Study) are not aware of their disease. Results are similar to those reported in previous studies in our context [37] and in similar settings in Latin America [38].

### 4.3. Public Health Relevance and Implications

As the developed risk score is simple, it does not require a blood test or laboratory services, and it might be easily implemented in clinical practice. Moreover, because our score asks for general information in the form of age and diabetes in first-degree relatives and is complemented by a simple anthropometric measure of waist, there is potential for the score to be self-administered.

According to our results, any patient aged 55 years and above and having at least one first-degree relative with T2DM has greater probability of having undiagnosed diabetes but also is at risk of developing diabetes in the future. In addition, a greater central obesity, that is, 100 cm or more, independent of the other terms of the score is alone a good predictor of diabetes as reported in previous studies [23]. Our algorithm included waist circumference instead of body mass index as other risk scores, providing a better indicator of accumulation of visceral fat and metabolic dysfunction in our context [39].

Recently, the Peruvian Ministry of Health has published the Guide of Clinical Practice for Diagnosis, Treatment and Control of Diabetes Mellitus in Primary Care [40] and only recommends screening in general population with plasma glucose among adults between 40 and 70 years with obesity or overweight as suggested by the American Diabetes Association [6]. As in other LMIC, plasma glucose is not always available in primary care, especially in semiurban and rural areas; therefore, a major challenge to be overcome in many countries is the implementation of a simple, fast, and laboratory-free based screening method. Moreover, within the Peruvian context, no risk score has been proposed as part of the aforementioned guide. Thus, our algorithm might fill a gap to facilitate further specialized assessment of high risk individuals for diabetes, an approach that may be of utility to various other countries facing similar challenges.

### 4.4. Strengths and Limitations

The strengths of this study include the use of a national population-based survey, including urban and rural areas across major geographical regions, to develop the Peruvian diabetes risk score, as well as its validation using bootstrap but also an independent longitudinal cohort study. Additionally, it is only based on three variables ensuring its simplicity to be used and implemented. However, the study has also some limitations. First, we have utilized fasting plasma glucose as the gold standard for diagnosing diabetes instead of an oral glucose tolerance test (OGTT). Although the OGTT is more sensitive and specific than the fasting plasma glucose, more cases would have been detected with the overload of glucose; it is rarely performed as part of the routine clinical practice. Second, the CRONICAS Cohort Study did not include information from the Amazon rainforest as did the ENINBSC survey. When a sensitivity analysis was performed excluding individuals from the jungle from ENINBSC data, results were similar to those presented in this manuscript (data not shown). In addition, the score was created using a national survey to be applicable to the entire Peruvian population. Third, some variables were not assessed in our logistic regression model such as dietary intake or history of gestational diabetes as such data was not available. As a result, some caution should be made when our algorithm is compared to other risk scores. Fourth, our model is based on the idea of risk stratification instead of individualisation [41]; for instance, variables were categorized instead of being preserved as numerical. Nevertheless, the performance of our score did not change when age and waist circumference were treated as numerical variables (data not shown). Moreover, our idea was to develop a simple and easily applicable score instead of a complex algorithm for predicting undiagnosed and incident diabetes. Finally, as other diabetes risk scores, the model warrants further scrutiny before it can be used in other populations.

## 5. Conclusions

The Peruvian diabetes risk score, built using age, self-reported diabetes in first-degree relatives, and waist circumference, proves to be a simple pragmatic screening tool for undiagnosed and incident cases of diabetes in Peru. This experience in generating such simple, easy-to-use approaches for the identification of T2DM can serve to inform other similar LMIC efforts who are on early stages of diabetes prevention. This tool, due to its simplicity, can facilitate various initiatives oriented to introduce and scale up early preventative and management strategies on a wider scale.

## Supplementary Material

As 371 (12.3%) subjects were not re-contacted during follow-up, we have conducted a comparison between participants included and excluded from longitudinal analysis.

## Figures and Tables

**Table 1:** Sociodemographic characteristics of participants without history of type 2 diabetes in the two involved studies.

	ENINBSC study	CRONICAS study
	(*n* = 2,472)	(*n* = 2,945)
*Demographic variables*		
Sex (% females)	1,209 (48.9%)	1,500 (50.9%)
Age (mean (SD))	50.5 (12.1)	55.3 (12.7)
Education in years (mean (SD))	7.8 (4.9)	8.0 (4.9)
*Behavioural variables*		
Current smoking (%)	391 (15.9%)	369 (11.5%)
Alcohol use (%)	2,323 (94.1%)	1,600 (54.3%)
Family history of diabetes (%)	268 (11.2%)	351 (11.9%)
Physical activity (% low level)	606 (24.5%)	938 (31.9%)
*Anthropometric measures*		
Body mass index (mean (SD))	25.7 (4.5)	27.6 (4.6)
Waist circumference (mean (SD))	91.0 (11.4)	91.5 (11.0)
Waist-to-height ratio (mean (SD))	0.58 (0.08)	0.59 (0.07)
Systolic blood pressure (mean (SD))	114.5 (18.5)	117.2 (18.9)
Diastolic blood pressure (mean (SD))	71.1 (11.9)	73.4 (11.1)
Hypertension (%)	579 (23.8%)	705 (24.0%)
Total cholesterol (mean (SD))	174.2 (36.9)	199.7 (39.6)
HDL-cholesterol (mean (SD))	43.5 (5.3)	41.7 (11.5)

SD: standard deviation and HDL: high-density lipoprotein.

Results may not add due to missing values.

**Table 2:** Risk factors and beta coefficients for undiagnosed diabetes: final regression model using CENAN database (*n* = 2,367).

	Bivariate model		Final model^*∗*^		Score
	Coefficient (SE)	OR (95% CI)	Coefficient (SE)	OR (95% CI)	
*Sex *					
Male (versus female)	−0.39 (0.30)	0.68 (0.38–1.21)			
*Age*					
≥55 (versus <55 years)	0.72 (0.29)	2.05 (1.16–3.64)	0.61 (0.18)	1.85 (1.30–2.63)	1 (versus 0)
*Current smoking*					
Current (versus never/former smoker)	−1.06 (0.60)	0.34 (0.11–1.12)			
*Alcohol user *					
User (versus never user)	0.38 (0.74)	1.46 (0.34–6.27)			
*Diabetes in relatives *					
Yes (versus no)	1.06 (0.34)	2.90 (1.48–5.66)	0.85 (0.42)	2.34 (1.04–5.31)	1 (versus 0)
*Physical activity*					
Low (versus moderate/high levels)	0.80 (0.30)	2.24 (1.25–4.01)			
*Body mass index*					
Overweight (versus normal)	0.07 (0.35)	1.07 (0.54–2.13)			
Obese (versus normal)	0.80 (0.36)	2.23 (1.11–4.49)			
*Waist circumference*					
90.0 to <99.9 cm (versus <90 cm)	0.66 (0.38)	1.93 (0.91–4.10)	0.74 (0.33)	2.09 (1.09–4.02)	1 (versus 0)
100+ cm (versus <90 cm)	1.41 (0.37)	4.10 (1.99–8.44)	1.40 (0.23)	4.07 (2.60–6.40)	2 (versus 0)
*Waist-to-height ratio*					
0.50–0.59 (versus <0.50)	0.34 (0.63)	1.41 (0.41–4.86)			
0.60–0.69 (versus <0.50)	1.09 (0.62)	2.97 (0.88–10.0)			
0.70+ (versus <0.50)	1.58 (0.68)	4.84 (1.27–18.5)			
*Hypertension*					
Yes (versus no)	0.52 (0.31)	1.68 (0.91–3.09)			

^*∗*^The model was created using backward elimination from the initial full model until we reached a final model with statistically significant covariates.

**Table 3:** Performance of different cut-points for detecting undiagnosed type 2 diabetes in the development database.

Total score	At high risk^*∗*^	Sensitivity	Specificity	PPV	NPV	Correctly classified	LR+	LR−
≥1	69.8%	93.5%	30.6%	2.6%	99.6%	31.8%	1.34	0.21
≥2	34.9%	69.6%	65.8%	3.9%	99.1%	65.9%	2.04	0.46
≥3	11.0%	30.4%	89.4%	5.4%	98.5%	88.3%	2.87	0.78
≥4	1.3%	2.2%	98.7%	3.2%	98.1%	96.8%	1.68	0.99

PPV: positive predictive value; NPV: negative predictive value; LR+: positive likelihood ratio; LR−: negative likelihood ratio.

^*∗*^Those at high risk are the proportion of participants over the total score.

**Table 4:** Performance of different diabetes risk scores compared to Peruvian diabetes risk score using the CRONICAS study (validation sample).

Method (proposed cutoff)	# of variables	AUC	Sensitivity	Specificity	PPV	NPV	LR+	LR−
Brazilian risk score (≥18)	3	0.65	66.7%	61.9%	4.9%	98.4%	1.75	0.54
Qingdao risk score (≥17 and ≥14)^*∗*^	4	0.58	83.3%	33.3%	3.6%	98.5%	1.25	0.50
Indian risk score (≥21)	5	0.54	94.0%	15.5%	3.1%	98.9%	1.11	0.39
Kuwaiti risk score (≥32)	4	0.62	45.2%	78.4%	5.8%	98.0%	2.09	0.70
Patient self-assessment score (≥5)	6	0.64	61.4%	66.8%	5.1%	98.3%	1.85	0.58
Rotterdam risk score (≥36)	6	0.55	94.0%	16.8%	3.2%	99.0%	1.13	0.35
Peruvian risk score (≥2)	3	0.68	70.2%	58.9%	4.8%	98.5%	1.71	0.51

AUC: area under the ROC curve; PPV: positive predictive value; NPV: negative predictive value; LR+: positive likelihood ratio; LR−: negative likelihood ratio.

^*∗*^Different cutoffs for males (≥17) and females (≥14).
