# Supplementary material for: Development and Validation of a Simple Risk Score for Undiagnosed Type 2 Diabetes in a Resource-Constrained Setting
Source: J Diabetes Res. 2016 Sep 4;2016:8790235. doi: 10.1155/2016/8790235 (PMC5027039; doi:10.1155/2016/8790235)
Supplement: Supplementary file 1 — As 371 (12.3%) subjects were not re-contacted during follow-up, we have conducted a comparison between participants included and excluded from longitudinal analysis. [file 8790235.f1.docx]

# ONLINE SUPPLEMENT

**E-Table 1:** Baseline characteristics among those included and excluded from longitudinal assessment of the risk score

|  | **CRONICAS Cohort Study** | |
| --- | --- | --- |
|  | **Included**  **(n = 2,577)** | **Excluded**  **(n = 371)** |
| **Demographic variables** |  |  |
| Sex (% females) | 1,309 (50.8%) | 191 (51.5%) |
| Age [mean (SD)] | 55.0 (12.6) | 57.1 (13.6) |
| Education in years [mean (SD)] | 8.1 (4.9) | 7.4 (5.2) |
| **Behavioural variables** |  |  |
| Current smoking (%) | 323 (12.5%) | 45 (12.1%) |
| Alcohol use (%) | 1,387 (53.8%) | 213 (57.4%) |
| Family history of diabetes (%) | 315 (12.2%) | 36 (9.7%) |
| Physical activity (% low level) | 805 (31.2%) | 133 (35.8%) |
| **Anthropometric measures** |  |  |
| Body mass index [mean (SD)] | 27.7 (4.5) | 26.8 (4.6) |
| Waist circumference [mean (SD)] | 91.8 (10.8) | 89.9 (12.0) |
| Waist-to-height ratio [mean (SD)] | 0.59 (0.07) | 0.58 (0.08) |
| Systolic blood pressure [mean (SD)] | 116.8 (18.5) | 119.8 (21.1) |
| Diastolic blood pressure [mean (SD)] | 73.1 (11.0) | 75.0 (11.5) |
| Hypertension (%) | 611 (23.7%) | 95 (25.6%) |
| Total cholesterol [mean (SD)] | 200 (39.6) | 197 (39.9) |
| HDL cholesterol [mean (SD)] | 41.6 (11.6) | 42.1 (11.2) |
